# Microstructure, Fractography, and Mechanical Properties of Hardox 500 Steel TIG-Welded Joints by Using Different Filler Weld Wires

**DOI:** 10.3390/ma15228196

**Published:** 2022-11-18

**Authors:** Zhaoyang Zuo, Ma Haowei, Mahdireza Yarigarravesh, Amir Hossein Assari, Moslem Tayyebi, Morteza Tayebi, Bejan Hamawandi

**Affiliations:** 1 School of Mechanical Engineering, Xijing University, Xi’an 710123, Shaanxi, China; 2 Department of Mechanical Engineering, Faculty of Engineering, University of Malaya, Kuala Lumpur 50603, Malaysia; 3 Department of Civil Engineering, Adjunct Faculty, Sharif University of Technology, Tehran 11155-8639, Iran; 4 Department of Material Engineering, Sahand University of Technology, Tabriz 551335-1996, Iran; 5 Department of Materials Science and Engineering, Shiraz University of Technology, Modarres Blvd., Shiraz 71557-13876, Iran; 6 Young Researchers and Elites Club, Science and Research Branch, Islamic Azad University, Tehran 14778-93855, Iran; 7 Department of Applied Physics, KTH Royal Institute of Technology, SE-106 91 Stockholm, Sweden

**Keywords:** acicular ferrite, Charpy impact test, filler metals, fracture surfaces, steel, tungsten inert gas welding

## Abstract

This paper deals with the effects of three low-carbon steel filler metals consisting of ferritic and austenitic phases on the weld joints of the tungsten inert gas (TIG) welding of Hardox 500 steel. The correlation between the microstructure and mechanical properties of the weld joints was investigated. For this purpose, macro and microstructure were examined, and then microhardness, tensile, impact, and fracture toughness tests were carried out to analyze the mechanical properties of joints. The results of optical microscopy (OM) images showed that the weld zones (WZ) of all three welds were composed of different ferritic morphologies, including allotriomorphic ferrite, Widmanstätten ferrite, and acicular ferrite, whereas the morphology of the heat-affected zone (HAZ) showed the various microstructures containing mostly ferrite and pearlite phases. Further, based on mechanical tests, the second filler with ferritic microstructure represented better elongation, yield strength, ultimate tensile strength, impact toughness, and fracture toughness due to having a higher amount of acicular ferrite phase compared to the weld joints concerning the other fillers consisting of austenitic and ferritic-austenitic. However, scanning electron microscopy (SEM) images on the fracture surfaces of the tensile test showed a ductile-type fracture with a large number of deep and shallow voids while on the fracture surfaces resulting from the Charpy impact tests and both ductile and cleavage modes of fracture took place, indicating the initiation and propagation of cracks, respectively. The presence of acicular ferrite as a soft phase that impedes the dislocation pile-up brings about the ductile mode of fracture while inclusions may cause stress concentration, thus producing cleavage surfaces.

## 1. Introduction

Taking into account recent developments in welding, there are now many types of welding processes that are extensively used by manufacturers, depending on the advantages and disadvantages of each process [1,2,3]. These processes are mainly divided into pressure and non-pressure welding processes, both of which are sub-divided into fusion and non-fusion processes [1,4,5,6].

The non-pressure fusion welding process uses a fusion of the base metal (BM) to make the weld, which can be either homogenous or heterogeneous [5,7,8]. The three major types of this process are gas welding, arc welding, and high-energy beam welding in which the heat sources are a gas flame, an electric arc, and a high-energy beam, respectively. The power density of the heat sources increases from a gas flame to an electric arc and a high-energy beam. As the power density of the heat source increases, the required heat input to the workpiece for welding decreases, which indicates the increasing penetration, welding speed, weld quality, and equipment cost; on the other hand, decreasing damages to the workpiece, including weakening and distortion [9]. 

One of the main disadvantages of welding processes is the dissolution of nitrogen, oxygen, and hydrogen gases in the weld metal which affects the soundness of the resultant weld. These elements possibly stem from the moisture or dirt on the surface of a workpiece, air, and consumables, such as the shielding gas and flux [10,11]. In most cases, these three elements have detrimental effects on welds. Nevertheless, they may have positive effects as nitrogen may increase strength and oxygen also increases the toughness when the content of the acicular ferrite in the weld metal increases with the oxygen content of the weld [9,12].

TIG welding is one of the homogenous non-pressure fusion welding processes in which a non-consumable tungsten electrode is used to establish an arc to melt and join metals, and an inert gas, such as helium or argon, is used as the shielding gas. Using an inert gas enables this process to be protected from the air [2,13].

Considering the limited heat inputs of the TIG welding process, this process is widely used for joining thin sections. There is a relatively wide variety in the amount of fusion between the BM and the filler metal, since the feeding rate of the filler metal is nearly independent of the welding current. This advantage enables the operators to have better control of dilution and energy input to the weld without changing the size of the weld. However, the brittle tungsten inclusions in the weld metal are one of the drawbacks of this process, which is triggered by the melting of the tungsten electrode due to excessive welding currents [9]. 

Steels are the most common materials used in industries because of their versatility and cost [14]. Among their wide range from plain carbon to high alloy steels, low-alloy wear-resistant steels, and above all Hardox steels, have recently attracted significant interest because of their good weldability, machinability, high mechanical properties, favorable wear-resistant, relatively low crack sensitivity, and good ductility [15,16]. These advantages allow these materials to be used as elements of construction machinery, such as beds of dump trucks and the buckets of excavators and loaders, in which these elements are subject to abrasive materials, including soil and gravel [17,18,19,20]. 

As Hardox steels are mainly considered to be low alloy steels, their microstructure and properties may differ based on the content of carbon and alloying elements, such as Ni, Mn, Mo, and Cr, which somewhat affect their durability, hardness, and hardenability [17]. This variety in compositions and structures complicates the investigation across the weld zone (WZ) [16,20,21,22]. For example, it has been reported that low hydrogen ferritic steel filler leads to better transverse tensile and fatigue properties, whereas austenitic stainless steel filler results in better impact toughness [21,22]. Additionally, as reported by Sharma and Shahi [23], joint welded using filler metal containing Cr and Mo with Nb, Ti, Al, V, Cu, and N as micro-alloying additions represented a weld metal, wherein martensitic refinement occurred and had the highest microhardness of about 400 HV. Since these alloying elements lead to the formation of carbides and inclusion, detailed knowledge of the distribution of impurities and structures may result in a better understanding of the probable crack formation in terms of their origin and mechanisms. 

The popular abrasive-wear resistant steel Hardox 400 is usually characterized by good weldability [24]. Nevertheless, the thermal processes of welding lead to the degradation of microstructures in HAZ by creating an unhardened layer; this results in significant changes of hardness [25]. These phenomena are often accompanied by not only welding but also the processing and forming operations of the constructional elements of the filler metals. The unfavorable structures and hardness levels which occur in welded joints of low-alloy, high-strength steels can be greatly changed by filler metal compositions [26]. Based on the previous studies [25,27], it can be concluded that it is worth complementing the issues related to making and optimizing the properties of welded joints of Hardox 500 steel. Plus, this is often motivated by the adverse opinion about the weldability of this steel, which usually results in the resignation of its welding or in replacing it with another grade with lower strength but better weldability [28,29,30,31,32,33,34,35,36,37,38,39,40,41,42,43,44,45,46,47]. According to the results of many studies related to the chemical and structural properties of low-alloy martensitic steels, as a whole, it can be stated that Hardox steels show good weldability but depending on welding conditions, susceptibility to cracking is achieved [48]. Nevertheless, it is confirmed by research works that practically they cannot be joined by welding. The most frequently observed problems with the weldability of the Hardox 500 are its susceptibility to the brittle cracking of the welded joints and the wide zones of lower hardness in comparison to the base material [49]. 

As mentioned earlier, Hardox steels are usually used in construction machinery which may experience failure in a workplace. Therefore, welding these steels is of importance. There are different types of filler metals which, depending on their chemical compositions and welding conditions, may result in various microstructures. These microstructures play an important role in determining the final properties. Acicular ferrite, for example, results in desirable mechanical properties. Hence, different filler metals should be used in the weld joints of 500 Hardox steel to investigate whether the brittle cracking can be avoided and whether favorable properties can be obtained or not. For this purpose, the present paper aims to examine the TIG welding of Hardox 500 steel by using three low-carbon steel fillers for controlling the microstructure and mechanical properties. The microstructural evolution of the weld zone (WZ), as well as the heat-affected zone (HAZ), were studied for each of the weldments. Special attention was paid to evaluating the effects of each filler on the mechanical properties of the WZ through microhardness, Charpy impact toughness, plane-stress fracture toughness, and tensile tests. 

## 2. Materials and Methods

### 2.1. Materials

In the present work, the Hardox 500 steel plate (120 × 60 × 10 mm^3^) was used as a BM, and three low carbon steel wires (2.4 mm diameter) were used as filler metals consisting of ferritic (ER80S-G), austenitic (ER80S-B2), and ferritic + austenitic (ER80S-Ni1). The chemical composition of these materials was measured by XRF and is given in Table 1, according to ASTM standards.

### 2.2. Welding Process

To weld the workpieces, TIG welding (Everlast PowerTIG 325EXT-Sahand-Tabriz-Iran) was utilized under argon gas protection with a volume flow rate of 10 L/min. The diameter of the electrode was 2.5 mm and its distance to the steel plates was 2 mm during TIG welding. Additionally, the traveling speed was 150 mm/min. Before welding, the surfaces and edges of the BM plate, as well as the filler metals, were cleaned to avoid the presence of contamination, such as oxide, oil, and moisture by which the weld defect is bound to form. 

**Figure 1:**
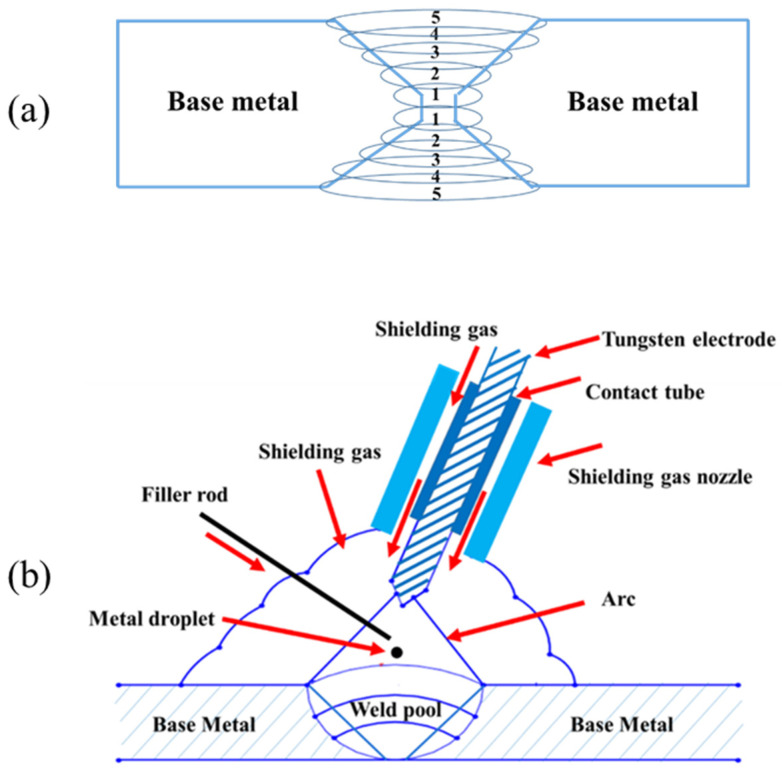
Schematic of (**a**) double V-groove weld and (**b**) the welding process.

The butt joint welding was carried out on the double V-groove joints (Figure 1). During the welding, the molten weld pool was held by gravity due to the flat position of the weld joint. Then, on each side of the joints, five passes of welding were applied (ten passes in total). Additionally, in order to control the residual stress of weld, each welding pass was conducted in a reverse direction of the previous pass.

The arc voltage was about 11 V, by which the arc can be established and maintained. Further, the direct current (DC) was about 150 A. All these parameters were selected to minimize the risk of electrical shock during the welding. It should be noted that all welds were carried out in the same condition by using three different filler metals. 

### 2.3. Metallographic Preparation and Microscopy Analysis

To study the various microstructures across the welded joints, the samples were taken from different regions. Then, all samples were mounted in epoxy resin. After being ground on silicon carbide papers and polished with 3 µm Al_2_O_3_ solution, the samples were chemically etched for 30 s with 5% Nital (2 mL HNO_3_ + 98 mL C_2_H_6_O) solution. Thereafter, all the prepared samples were characterized by using the Olympus OM model PMG3™, the EOS SEM model CamScan MV2300™ (manufacturer: Electron Optic Services, Inc-Canada/Place of testing: Tabriz-Iran) and XRD apparatus type (XRD-6000, Shimadzu, Kyoto, Japan) with Cu Kα radiation (λ = 1.54056 Å). The measurement was set at 2θ in a range of 10° to 90°.

### 2.4. Microhardness Tests

Based upon ASTM-E 384 standard, the Vickers microhardness test was carried out using a Karl Frank GmbH-38536 (Manufacturer: Germany/Place of testing: Tabriz-Iran) apparatus under a load of 0.5 kg for 10 s across the BM zone, HAZ, and the WZ. It should be noted that at least five separate measurements were performed on each zone, with the mean data being reported. 

### 2.5. Tensile Tests 

**Figure 2:**
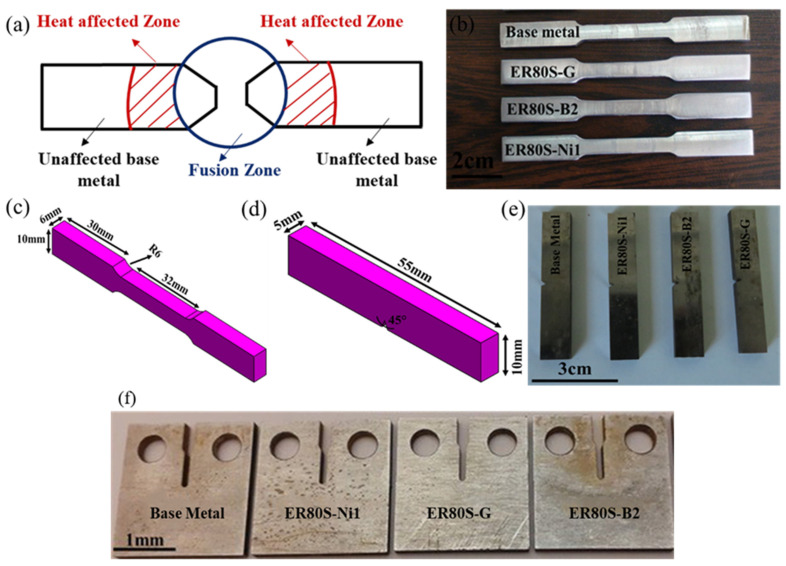
(**a**) Schematic of HAZ and fusion zone and samples of (**b**,**c**) tensile, (**d**,**e**) Charpy impact, and (**f**) fracture toughness tests.

To study the mechanical properties of the welded joints, as can be seen in Figure 2a, the samples were wire-cut based upon the ASTM E8M-04 standard in that the WZ, which represents the weakest region, was located in the gauge section (Figure 2b). Then, to minimize the stress concentration the surfaces of the wire-cut samples were magnetically ground. The uniaxial tensile tests were carried out at a strain rate of 1 × 10^−4^ s^−1^ at room temperature using an Instron tensile test machine. For each weldment, three tensile tests were performed to ensure the accuracy of the results. In addition, the fracture surfaces of each welded joint were examined by SEM.

### 2.6. Charpy Impact Test

First, the samples regarding each weldment were wire-cut following ASTM A370 from the weld, in that the V-notch is taken from the WZ, as shown in Figure 2d. Next, the Charpy V-notch impact toughness test was performed for all three weldments. Before applying the test, the surfaces of the wire-cut samples were magnetically ground to minimize the stress concentration. Thereafter, the test was conducted through an impact testing machine with 400 J capacity, according to the ASTM E23-12c standard. Due to the possible errors during the impact test, for each weldment, three samples were tested at ambient temperature. The fracture surfaces were characterized by using SEM.

### 2.7. Plane-Stress Fracture Toughness Test

To measure the toughness of welds, the samples were wire-cut, as depicted in Figure 2f. Then, they were tensioned using the tensile test machine. At the same time, the crack opening displacement (COD) gages were used to evaluate the crack length during loading. Afterward, the monitored values were reported. Moreover, the fracture surfaces of each sample were examined by SEM. 

## 3. Results and Discussion

### 3.1. Macrostructure Examination

**Figure 3:**
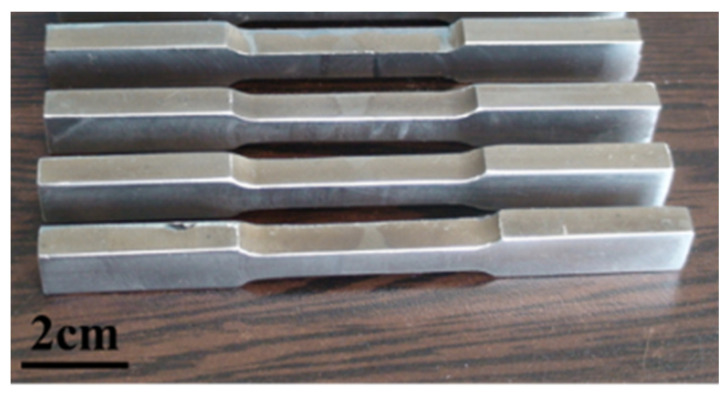
Macroscopic micrographs of the tensile test specimen.

Figure 3 shows macroscopic micrographs of the welded samples. Visual observation of the samples indicated that the penetration and fusion to root were achieved and the welding process performed desirably, without cracks and undercuts. 

### 3.2. Microstructure Characterization 

**Figure 4:**
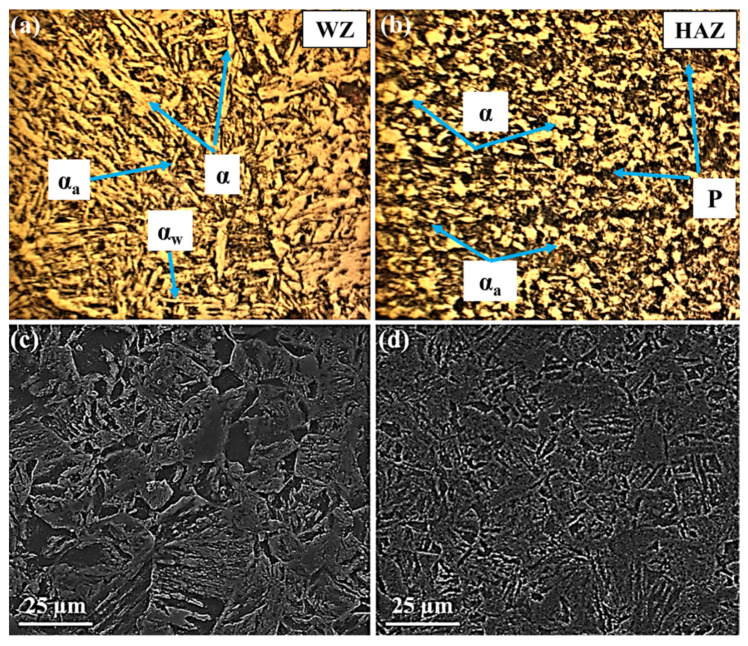
OM and SEM images showing the microstructures of the first weld: (**a**,**c**) weld zone (WZ), (**b**,**d**) heat-affected zone (HAZ).

**Figure 5:**
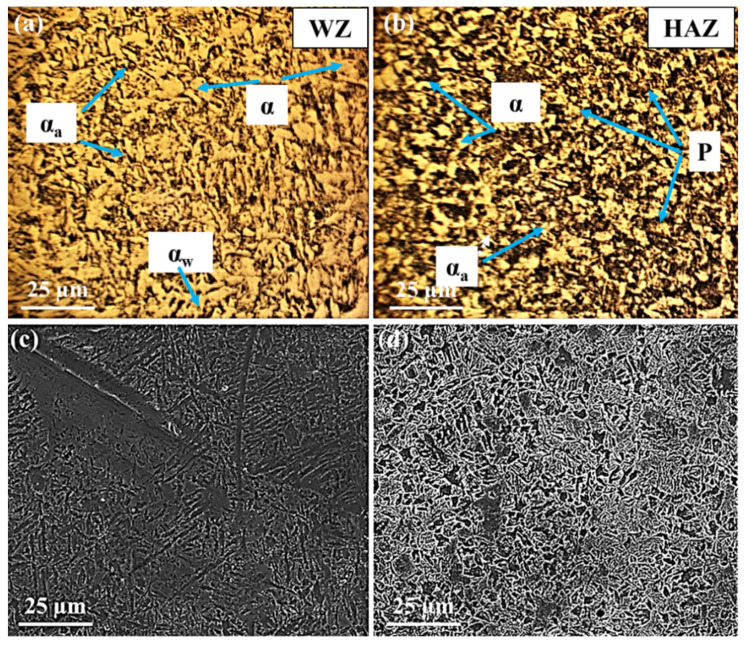
OM and SEM images showing the microstructures of the second weld: (**a**,**c**) weld zone (WZ), (**b**,**d**) heat-affected zone (HAZ).

**Figure 6:**
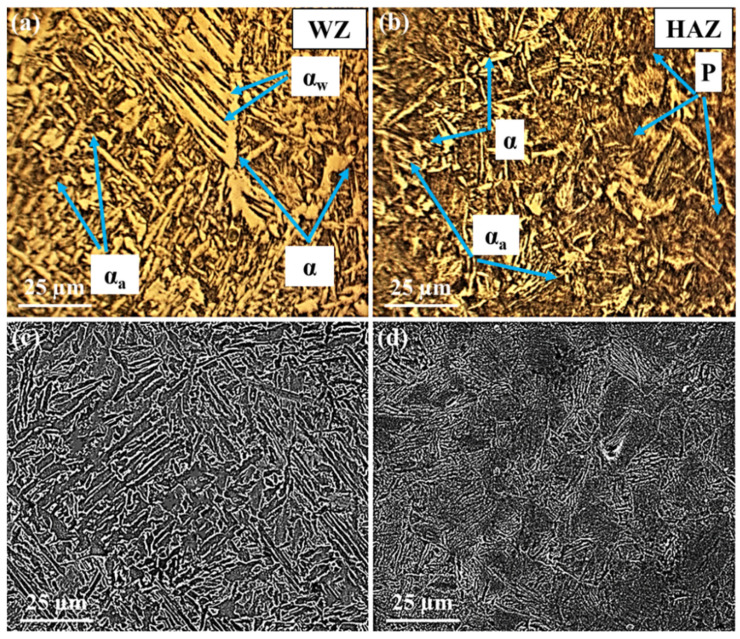
OM and SEM images showing the microstructures of the third weld: (**a**,**c**) weld zone (WZ), (**b**,**d**) heat-affected zone (HAZ).

Figure 4, Figure 5 and Figure 6 display the variation of structures in the WZ and HAZ. In the case of BM, it is generally known that its microstructure represents the martensite structure [17,50,51].

Regarding the WZ microstructure, different ferrite morphologies, including allotriomorphic ferrite (α), Widmanstätten ferrite (α_w_), and acicular ferrite (α_a_) can be seen in Figure 4, Figure 5 and Figure 6. It is believed that, as the weld cools from the liquid phase to ambient temperature, the δ-ferrite changes to austenite during a solid-state transformation, followed by allotriomorphic ferrite (α), which is likely to be the first phase to form on cooling the austenite grains below the Ae_3_ temperature. The grain boundaries of columnar austenite are easy diffusion paths for these ferrite layers to nucleate and grow. However, further undercooling, which increases the driving force for transformation and decreases the diffusivity, probably limiting the growth of allotriomorphic ferrite. Then, Widmanstätten ferrite is likely to grow in the remaining austenite. Widmanstätten ferrite plates lengthen at a constant rate because of their plate shape and the partitioning of carbon at the sides of its growing plate. However, it is generally agreed that the formation of Widmanstätten ferrite plates is usually completed within a fraction of a second but most of them may be interfered with by plates of acicular ferrite [52,53]. 

The acicular ferrite is believed to nucleate at non-metallic particles dispersed within the weld. Therefore, as can be seen from Figure 5, the quantity of acicular ferrite in the WZ of the second filler is considerably more than that of the other fillers. This is because the second filler with a higher amount of sulfur (S) and manganese (Mn) represented more acicular ferrite. In fact, despite the different ferritic phases, the carbon of the WZ may be consumed by the formation of cementite and carbides, in so far as the nucleation of carbides may take place at the interface of ferritic phases and cementite particles. The carbon near cementite particles disappears gradually as the carbides grow. Among all alloying elements present in this research, Cr, Mo, V, W, and Ti are bound to form carbides, whereas Ni, Co, and Cu are unlikely to form carbide phases [52,53].

It is widely accepted that various inclusions can be found during steel welding, including Al_2_O_3_, spinel-type oxides (AO-B_2_O_3_), FeO, MnO, (FeMn)O, and more specifically manganese sulfide (MnS). Furthermore, it is suggested that a wide variety of oxides or other compounds, including SiO_2_ and a thin layer of MnS which partly covers the surface of the inclusions, are responsible for the development of microstructure, particularly the heterogeneous nucleation of acicular ferrite during cooling [23,52]. 

As can be seen in Figure 4, Figure 5 and Figure 6, allotriomorphic and Widmanstätten ferrite within the WZ of the first and the third fillers is larger than that of the second filler. This difference is attributed to the sensitivity of the growth kinetics of the microstructure of welds to carbon, in that the growth rates of allotriomorphic and Widmanstätten ferrite increase as the concentration of carbon declines [52]. 

**Figure 7:**
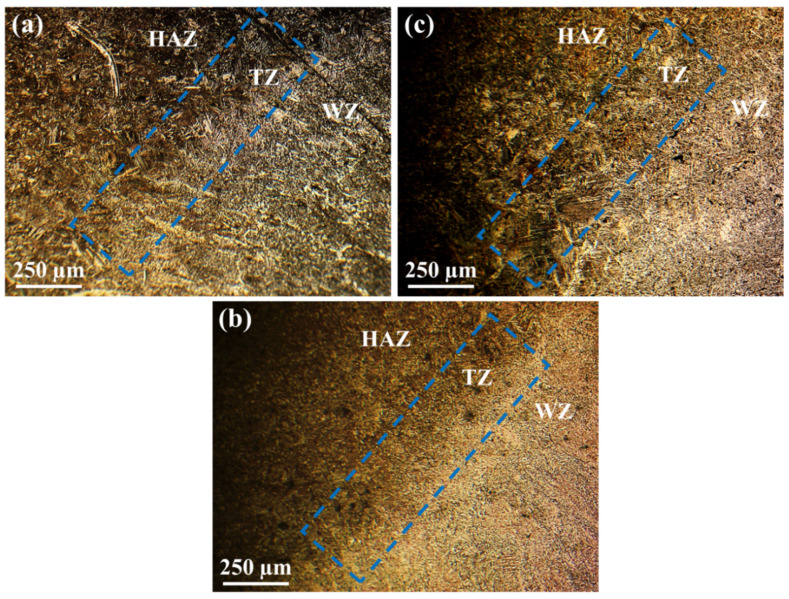
OM images showing the microstructures of the transition zone (TZ): (**a**) first weld, (**b**) second weld, and (**c**) the third zone.

Figure 7 shows an almost wide transition region between the WM and HAZ. It is almost certain that the formation of this region arises from local variations in composition and temperature. Since this region is surrounded by melting and the 100% solid region of the weld, several metallurgical phenomena may occur. Although this type of steel consists of low alloy content, the segregation of alloying and impurity elements are likely to take place at grain boundaries during processing. In addition, there possibly be localized melting temperatures at the grain boundaries in which liquation may occur. Moreover, the dissolution of carbide particles near HAZ is believed to lower its melting point. Finally, if these carbides and grain boundaries cool, residual stresses and liquation cracks appear in the transition region [52]. 

Concerning the HAZ, the microstructures contain ferrite and pearlite phases, as well as cementite and carbide particles, as represented in Figure 4, Figure 5 and Figure 6. These microstructural variations are brought by the non-equilibrium rates of heating and cooling during welding. Although the source of heat causes the melting of joints, the rate of heating and the peak temperature declines regularly with distance away from the WZ. It is known that the cooling may occur in a temperature range of 800–500 °C, in which the austenite is likely to decompose by solid-state transformation. This temperature range is different from the equilibrium temperatures Ae_1_ and Ae_3_; however, depending on the distance from the boundary of the weld metal, there would be coarse-grained and fine-grained austenite zones. The former tends to form adjacent to the WZ, where the temperature is above the Ac_3_ temperature of weldable steels. The latter may form further away from the WZ where the peak temperature decreases, resulting in the rather high carbon concentration of austenite, in which the solubility of carbon increases as the temperature decreases. In addition, the steel cannot often transform completely to austenite because of the peak temperature at a large distance from the WZ. In this case, the solubility of carbon in austenite is nearly in equilibrium with ferrite. Therefore, these austenite regions certainly decompose to both ferrite and cementite because the cooling rates are unlikely to induce martensitic transformation. The nucleation and formation of pro-eutectoid ferrite and cementite, before the nucleation of pearlite, may occur at the austenite grain boundaries. Thereafter, the pearlite nuclei probably takes place in these grain boundaries, as well as on inclusions. The relatively small amount of remaining austenite, which is certainly enriched in carbon probably transforms into pearlite. The nucleation of pearlite is two-fold. First, the nuclei of pearlite occurs on austenite grain boundaries, which are associated with pro-eutectoid ferrite and cementite and presumably does not have the lamellar structure due to the rates of slower cooling. Second, the nodules of pearlite can often nucleate on inclusions, especially in commercial steels. From Figure 4, Figure 5 and Figure 6, it can be seen that the pearlite concerning the weldment of the third filler is much bigger than that of other fillers. This may be related to two possible causes. First, the inclusions formed in the WZ may be pushed away during the welding process. Second, the formation of tungsten inclusions in weld. Considering the similar welding conditions for all three fillers, each of the mentioned reasons is responsible for the larger colonies of pearlite and even for the formation of acicular ferrite [52]. Apart from the mentioned structures, carbide precipitations appear to form by removing carbon from the supersaturated ferrite and the residual austenite. Indeed, Hardox 500 steel is known as low-alloyed plain carbon, and its alloying elements can often form different carbides, some of which may remain stable while the other carbides may be dissolved in austenite. It can be concluded that as the number of inclusion and carbides nucleation sites relative to the nucleation sites of austenite grain increases, the formation of acicular ferrite at the expense of Widmanstätten ferrite will certainly occur. 

### 3.3. Microhardness Measurement

**Figure 8:**
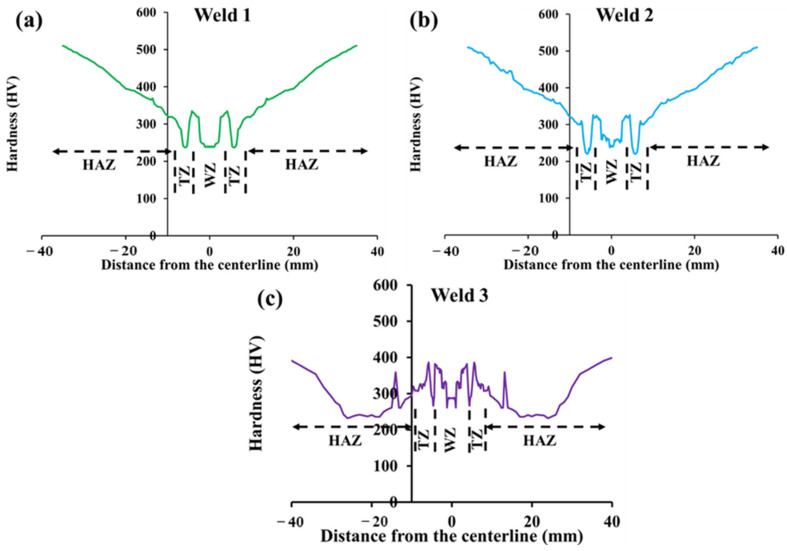
The profile of microhardness across the weldments: (**a**) first weld, (**b**) second weld, and (**c**) third weld.

Figure 8 displays the microhardness profile across the weldments for all three filler metals, to which the parts of Hardox steel 500 were welded. The values of the hardness of WZ regarding the first and the second filler metals are in the range of 220–330 HV, but these values of the third filler are in the range of 260–385 HV. These values are triggered by the existence of ferrite phases in the WZ. Additionally, the gradual rise of the hardness in this zone is probably related to the formation of the carbides, above all Cr_23_C_6_ [54]. 

**Figure 9:**
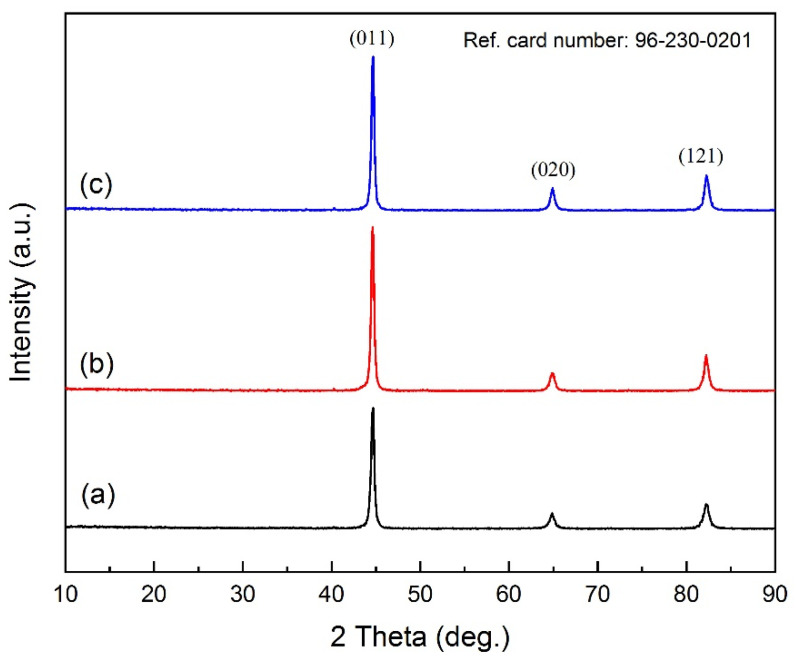
XRD patterns of welded samples. (**a**) the first weld, (**b**) the second weld, and (**c**) the thirdweld.

As can be seen, there is a drop in each of the hardness profiles, representing the transition zone. This zone, which is at the boundary of the WZ and heat-affected zone, is quite weak since it is subject to melting and solid regions during welding. Furthermore, the segregation of alloying elements and impurities mainly takes place in this region. Figure 9 shows XRD patterns of welded samples. Based on the figure, the peak of impurities cannot be recognized, which is due to the lower number of impurities. Thus, all of these reasons have detrimental effects on the hardness.

From Figure 8a,b, it can be observed that the HAZ of the first and the second filler metals show an upward trend. Since the cooling rate in the HAZ is relatively lower than that in the WZ, unlike the WZ, which is completely composed of ferrite phases with different morphology, pearlite is formed in HAZ, giving significant hardness. Thus, as the distance from the transition zone increases, the hardness grows modestly. This is because, in this zone, there is a combination of phases, including ferrite and pearlite. In addition, it is stated that the formation of niobium oxide during the welding process increases the microhardness [55]. Nonetheless, according to Figure 8c, after the transition zone, the hardness values experienced a decline from 390 to 261 HV. Thereafter, the values of hardness peaked at 360 HV. Afterward, it fell to 236 HV, then fluctuated, and was then followed by a sharp rise. It can be inferred that both acicular ferrite and allotriomorph ferrite have a profound effect on hardness values in WZ though the content and size of pearlite play important role in HAZ. Furthermore, the highest values of hardness that can be seen near base metal (BM) are caused by the decomposition of the martensitic phase to mostly ferritic phase and pearlitic phase. Depending on the cooling rate, it is believed that bainite is probable to form instead of pearlite, but based on the welding conditions in this work, pearlite was the second phase formed in HAZ region. Furthermore, near BM, there must be a higher amount of this phase compared to ferrite and that is responsible for the high values of hardness [56].

### 3.4. Tensile Tests

**Figure 10:**
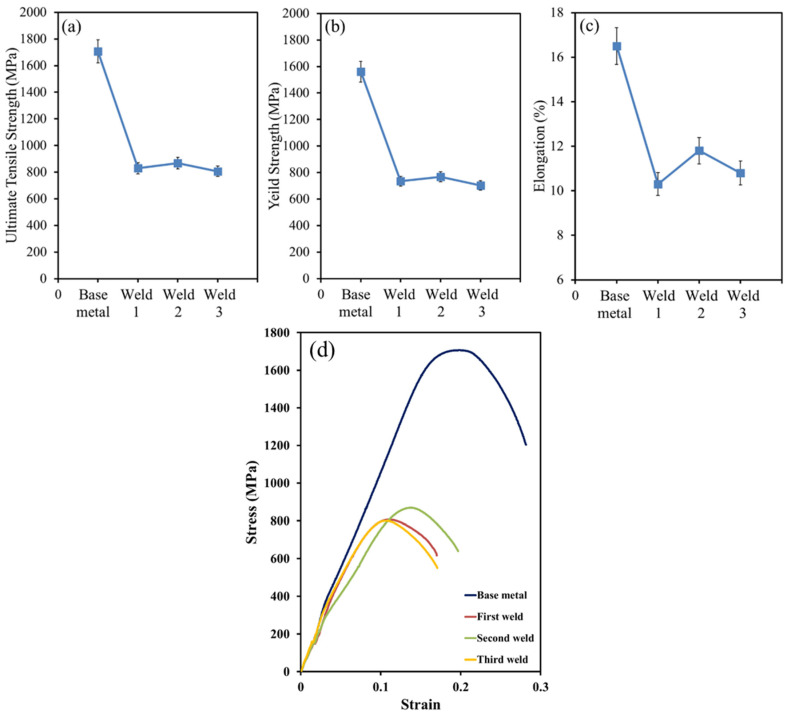
The mechanical properties of base metal and all three welds. (**a**) ultimate tensile strength (**b**) Yield strength (**c**) elongation (**d**) engineering stress-strain curve.

Figure 10 illustrate the tensile properties of BM and different welds using three filler metals. The ultimate tensile strength, yield strength, and elongation of BM were higher than those of all three welds. These considerable tensile properties are due to the microstructure of Hardox 500 steel, which is the martensite. However, the differences in the values of the welds are mainly attributed to the amount of acicular ferrite in the WZ rather than allotriomorphic and Widmanstätten ferrites. Acicular ferrite is extensively known as a desirable microstructure. It should be noted that the dispersion of fine alloy carbide in the temperature range of welding may markedly increase the strength, which confirms our observations.

As can be seen in Figure 4, Figure 5 and Figure 6, the higher percentage of acicular ferrite in the second weld compared to the other welds gives rise to these values. It is believed that an increase in yield stress of the weld regarding the second weld metal may stem from a fine dispersion of inclusions possibly delaying the cleavage fracture through the localized relaxation of stresses. Additionally, as reported by many researchers [50,52,57,58], inclusions rich in titanium stimulate the nucleation and formation of acicular ferrite. Based upon the chemical composition of all three filler metals, it is evident that the second filler has a higher percentage of titanium than the others by which a higher amount of acicular ferrite is expected to nucleate. 

**Figure 11:**
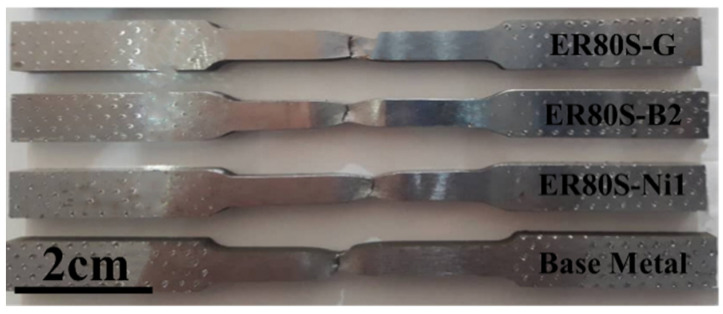
Tensile test specimen showing the necking and fractured regions.

From Figure 11, it can be seen that necking took place at fracture sections. Deformation caused by tensile test hardens the steel, and if at some point the loading rate at which it hardens is not sufficient to withstand the reduction in area, the deformation becomes focused, which thus leads to necking. Expectedly, a fracture occurred on the WZ, which is believed to be weaker than the HAZ and the BM. Furthermore, void nucleation takes place preferentially in the ferrite and then, during tension, the high density of voids often leads to localized necking [59,60]. 

**Figure 12:**
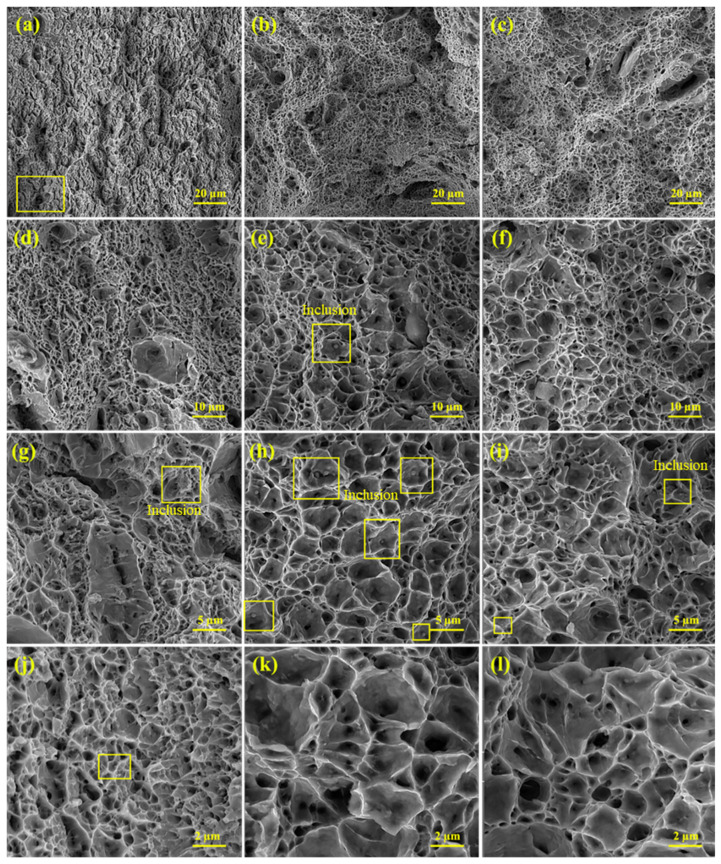
The fracture surfaces of tensile test pertaining to all three welds at different magnifications. (**a**,**d**,**g**,**j**) the first weld, (**b**,**e**,**h**,**k**) the second weld, and (**c**,**f**,**i**,**l**) the third weld.

Figure 12 display the fracture surfaces of WZs of three filler metals. All the weld joints are mainly composed of ductile fractures. Additionally, all surfaces indicate the cup and cone type of fracture. It is well-known that, in tension, this type of fracture usually involves the nucleation and growth of voids of which their coalescence results in ultimate failure. Plus, the presence of deep and shallow voids is visible at different magnifications. 

In some parts, the fragments of phases are likely to result from alloying elements as can be observed in Figure 12a–c. The alloying elements are believed not to fit favorably in the substitutional or interstitial sites within the lattice, and they tend to segregate to a greater free volume, especially grain boundaries. For example, the maximum reported values of the free energies of segregation for sulphur and phosphorus to be dissolved into iron are less than those for hydrogen. Furthermore, the microstructural factors, such as carbide precipitation, e.g., cementite or alloy carbides, either provide the sites for intergranular grain crack nuclei or cause interfacial separation. The latter takes place when the interfaces consist of Sb, As, Sn, or P [52].

Carbides, sulfide, or silicate inclusions produced by these elements across the WZ not only act as stress concentrators but also tend to initiate some transverse cracks. An illustration of this is the presence of brittle silicates and inclusions, especially alumina particles, in steels as a source of crack nuclei. Figure 12 displays several visible inclusions on fracture surfaces. The inclusions can probably be TiO_2_, Al_2_O_3_ FeO, MnO, and MnS. As mentioned earlier, these inclusions are often found during steel welding. These inclusions promote the nucleation and formation of acicular ferrite. It should be noted that the segregation of solute atoms preferentially to grain boundaries may easily provide crack nuclei [52]. Thus, such a scaly structure on fracture surfaces is created as a consequence of slips followed by de-cohesion and microcracks along determined crystallographic planes. This is because some of the ferritic phases, including Widmanstätten ferrite and acicular ferrite may form by displacive transformations, and this type of transformation involves the coordinated motion of atoms along determined crystallographic planes [53,61]. Further, there are some irregularities on interfacial surfaces, which are attributed to the cracks and mostly the ferrite phases.

### 3.5. Impact Toughness Analysis

**Figure 13:**
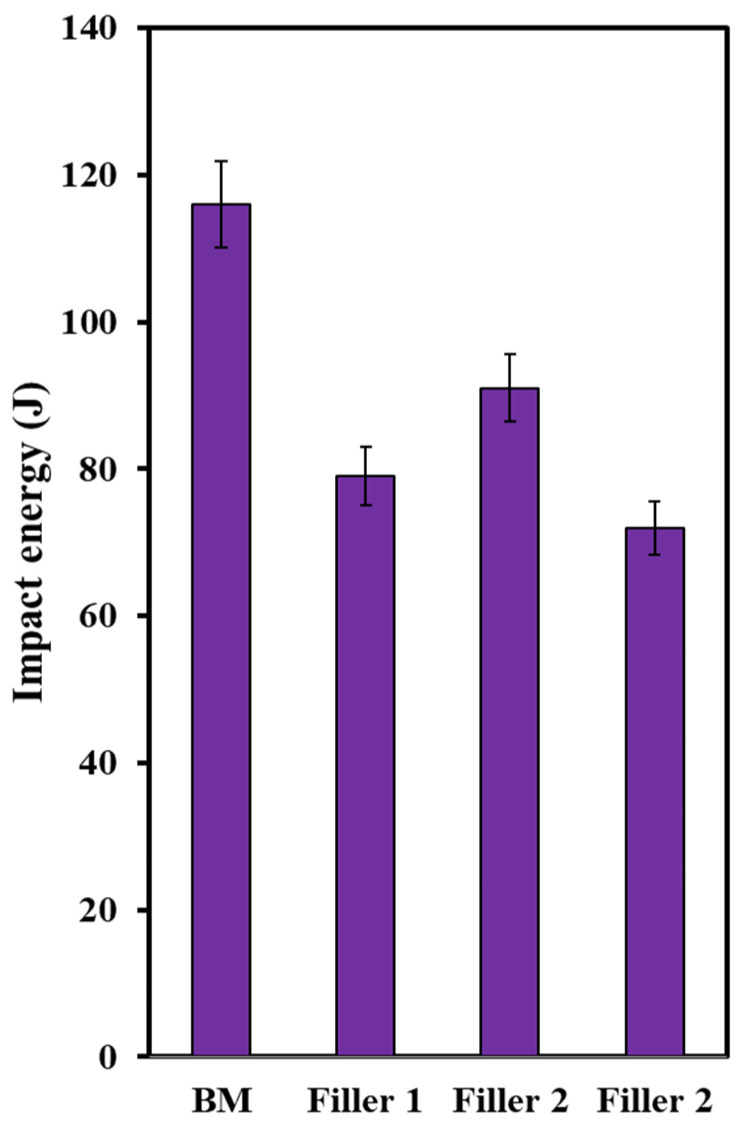
The values of Charpy impact energies.

The values of the energies are highlighted in Figure 13. The values are much higher than many other reported values [50,61,62] and, interestingly, the impact toughness of all three welds represents higher values than the base metal (BM). This is certainly because of the presence of acicular ferrite, which is highly considered a favorable phase showing desired toughness. The formation of this phase leads to interlocked microstructure, thus resulting in better toughness properties [63]. 

**Figure 14:**
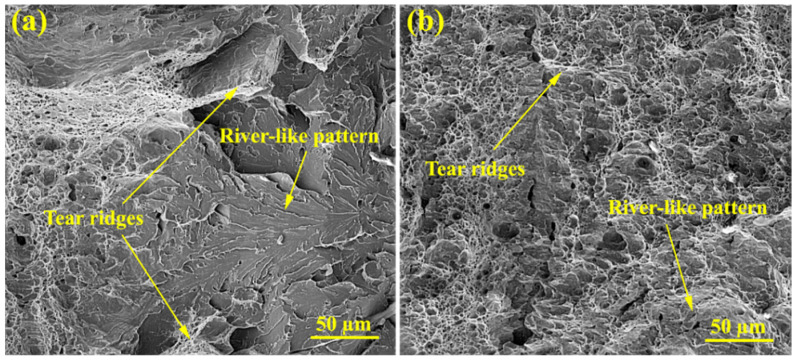
The fracture surfaces of the Charpy impact test of (**a**) the first weld and (**b**) the third weld.

From Figure 14 it can be seen that plastic deformation mainly exists on fracture surfaces with quite irregularities. Both ductile and brittle modes of fracture are represented on these surfaces through some well-known features, including the tearing ridges and river-like pattern, which indicate the relatively high values of impact toughness. 

**Figure 15:**
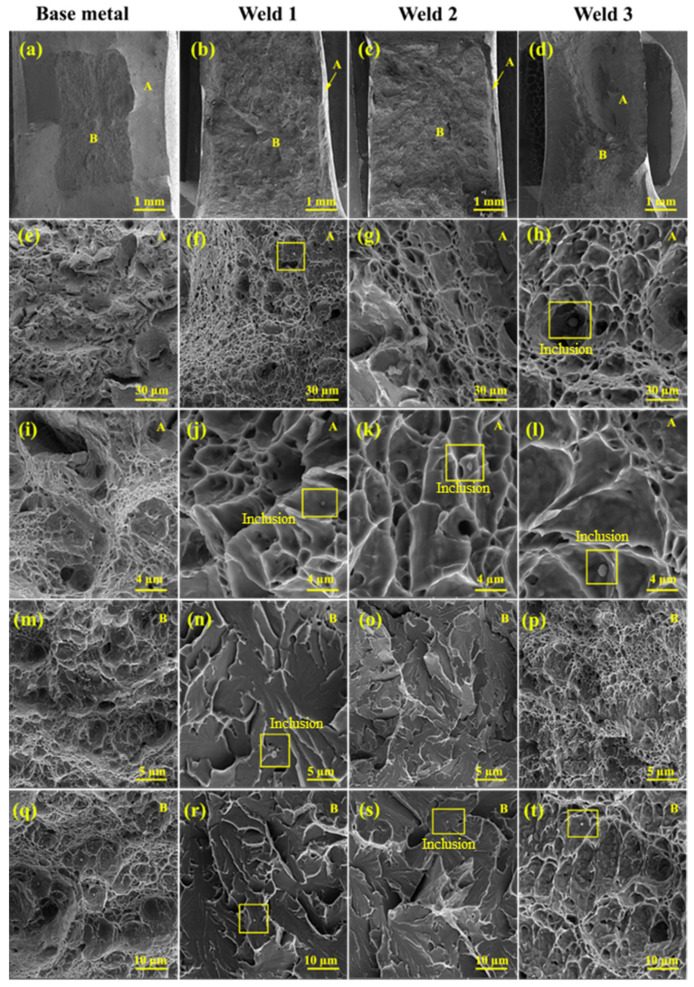
The fracture surfaces of Charpy impact test pertaining to all three welds at different magnifications (**a**–**t**).

Figure 15 display the impact fracture surface of all three welds. As the specimen was wire-cut in the transverse direction, the V-notch was located in the WZ. Both ductile and brittle modes of fracture occurred on these fracture surfaces. The ductile zones were on the edge of the fracture surface and are composed of dimples, whereas the brittle zones were on the center of these surfaces showing cleavage river patterns. However, concerning the third weld, the ductile mode of fracture was dominant. It is believed that the initiation of the crack brings about adjacent to the V-notch, which is in the ductile fracture zone and then the propagation of the crack takes place far from the V-notch, which is in the brittle zone. In fact, during the impact test, significant deformation occurs in the microstructure before work hardening by which the load increases. Finally, as the length of the crack increases, there is a marked drop in load, which then leads to the formation of the brittle zone with a river-like pattern, which is in good agreement with the observations of Korkmaz and Meran [64].

The values of impact energies are attributed to acicular ferrite, in which the glide of dislocations is easier. Since this phase is soft, the crack initiation can be impeded by releasing stress by forming dimples or voids, leading to the improved energy of crack initiation. It is believed that the coalescence of voids is responsible for the high values of impact energy. In addition, the visible irregularities, as well as the cavities with different sizes are triggered by the alloying elements. An illustration of this is the WZ of the first filler with a lower amount of C and Mn compared to the other two filler metals. This zone may hinder the initiation of microcracks on account of having few inclusions and carbides, even compared to the BM [23,64,65]. This feature confirms the larger portion of regions with the ductile mode of fracture, which is often caused by a high density of voids. Therefore, less stress concentration is expected to exist in the first weld, resulting in the higher values of impact energy. The same measured values for the second and the third filler metals may be due to the number of carbides and inclusions, as represented by the yellow dash circle in Figure 15.

### 3.6. Fracture Toughness Analysis

**Figure 16:**
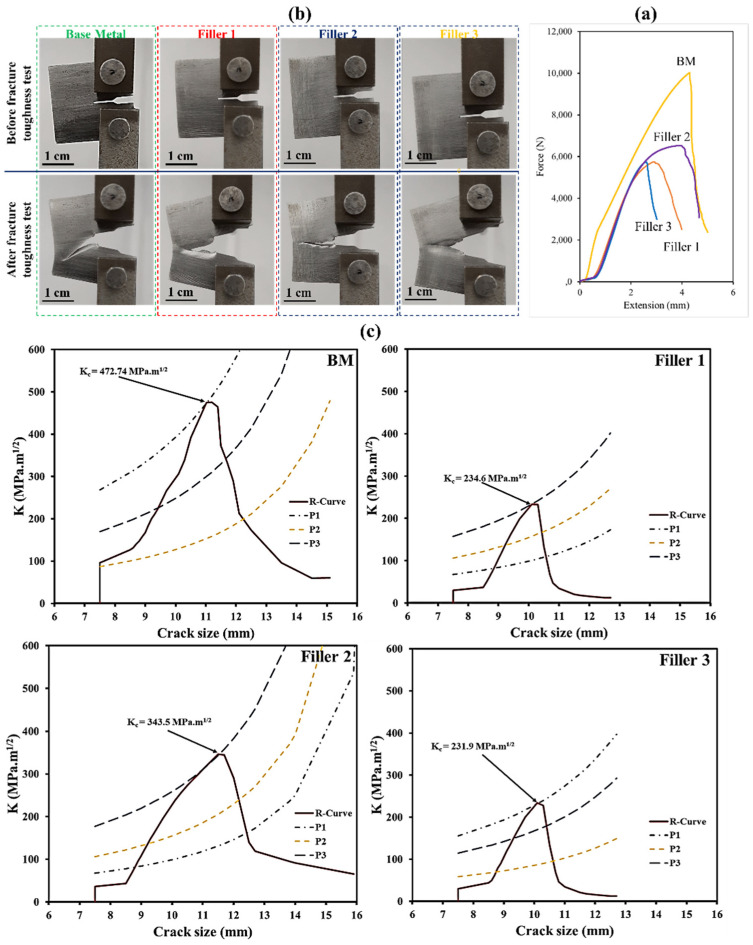
(**a**) Samples of plane stress fracture toughness and (**b**) force versus extension curve, (**c**) R-curves of base metal and weld zones.

Force versus crack length is presented in Figure 16b. These values were achieved after applying fracture toughness test. By using the equations below [66], based on the ASTM-E561 standard, the plane stress fracture toughness (*k_ri_*) values of base metal and all three welds were calculated.

(1) $$k_{r_{i}}=\frac{p_{i}}{b\sqrt{w}}\times \;f_{i}\left(\frac{a}{w}\right)\;\;$$

(2) $$f_{i}\left(\frac{a}{w}\right)=\left[\frac{2+\left(\frac{a}{w}\right)}{{\left(1-\left(\frac{a}{w}\right)\right)}^{\frac{3}{2}}}\right]\left[0.886+4.46\;\left(\frac{a}{w}\right)-13.32{\left(\frac{a}{w}\right)}^{2}+14.72{\left(\frac{a}{w}\right)}^{3}-5.6{\left(\frac{a}{w}\right)}^{4}\right]$$

Here, *a* is the crack length at various forces of *p_i_*. Additionally, *b* and *w* are the thickness and width of the compact-tension (CT) sample, respectively. By replacing the values of *a* and *w* at three different forces, the R-curves of base metal and three welds were obtained, as presented in Figure 16. Base metal possesses a higher value than three welds, which is attributed to its microstructure; in fact, a lath martensite microstructure offers excellent toughness. Regarding the three welds, in agreement with previous mechanical results, in which the second weld consisting of acicular ferrite showed better properties, this weld has better fracture toughness than the other welds. Accordingly, the first weld with more content of acicular ferrite than the third showed a higher value. 

**Figure 17:**
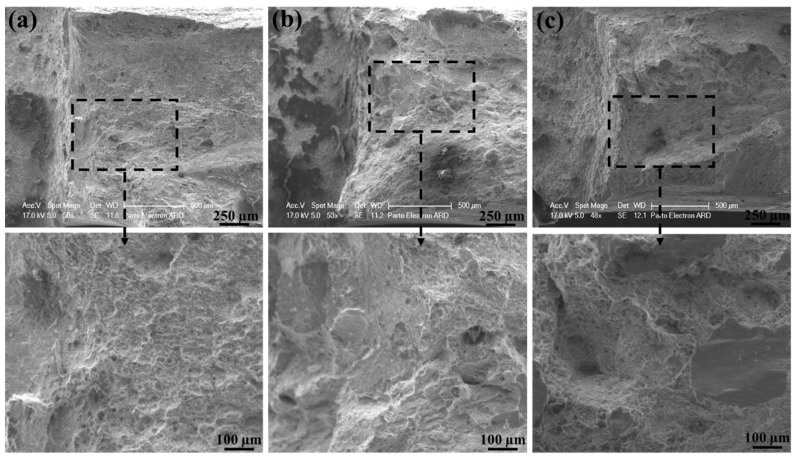
The fracture surfaces of fracture toughness test pertaining to all three welds at different magnifications. (**a**) the first weld; (**b**) the second weld; (**c**) the third weld.

From Figure 17, it can be seen that fracture surfaces are mainly composed of ductile mode with a large number of dimples. However, in some regions, tear ridges are evident and, in others, cleavage mode is observed. Even though ferrite phases are mainly responsible for ductile damage, it is generally believed that cleavage in low alloy steels is often initiated by either carbide cracking or dislocation pile-up. The latter usually takes place where inclusions exist. There are some common inclusions in structural steels, including TiC, TiO_2_, Al_2_O_3_, and MnS. As was detected earlier, MnS inclusions were prevalent in all three welds. During this test, when the samples are loaded, the inclusions are debonded. After that, on account of strong stress concentration, cleavage is initiated on some grains. However, from Figure 17, it is evident that there are not many cleavage regions, indicating that cleavage cracks were arrested by ferritic phases and could not propagate further. 

## 4. Conclusions

The Hardox 500 steel was TIG-welded by using three filler metals with different chemical compositions. To make a proper comparison of these three welds, the microstructures and mechanical properties of the welds were evaluated. Furthermore, to gain a deep understanding of the fracture modes regarding tensile and impact toughness tests, the fracture surfaces were investigated. The results are summarized as follows:

1. According to the OM images, carbides and inclusions not only promote the nucleation and growth of acicular ferrite but also slow down the growth of allotriomorphic and Widmanstätten ferrites. Moreover, the second weld metal with higher content of Mn and S certainly causes inclusions by which the nucleation of the acicular ferrite is likely to take place within the weld. The HAZ regions displayed ferrite and pearlite phases. During heating and cooling, the pro-eutectoid ferrite and the cementite nucleated and formed, followed by the nucleation and formation of pearlite nodules at the austenite grain boundaries.
2. The gradual rise of the microhardness in the WZ is attributed to carbides while the lowest values that occurred in the transition zone are due to the segregation of alloying elements and impurities. However, the modest increase in the HAZ is related to the existence of ferrite and particularly pearlite phases. However, the lower values of hardness in the third weld are due to the higher amount of acicular ferrite rather than allotriomorph ferrite.
3. The differences in the values of ultimate tensile stress, yield stress, and elongation are quite relevant to the amount of acicular ferrite in the WZ compared to other ferritic phases. Acicular ferrite was mostly observed in the weld joints of the second filler with ferritic microstructure. The fracture surfaces of tensile tests represented shallow and deep voids, which indicate ductile fracture mode. Acicular ferrite was responsible for this mode of fracture. Additionally, the fragment of phases, and cup and cone type of fracture may be produced by alloying elements and carbides.
4. The findings of the impact toughness and fracture toughness tests showed that the values of energies of all three welds are less than the toughness of BM but are acceptable. This means when the welding of Hardox 500 steels is needed, these filler metals and TIG welding can be applied. However, the higher value of the second weld is because of the presence of acicular ferrite and also due to the amount of alloying elements, such as Ti, C, S, and Mn. The weld joints of fillers with ferritic and austenitic microstructures represented the highest and the lowest values of mechanical properties due to the amount of acicular ferrite. This is because the weld zone (WZ) of the first filler mostly consisted of acicular ferrite, whereas the WZ of other fillers comprise allotriomorphic and Widmanstätten ferrites.
5. Finally, even though three different filler metals were used to weld Hardox steels, several potential limitations need to be considered. First, other types of filler metals with different chemical compositions can be examined. Second, the welding conditions can be altered to achieve better-quality of weld joints. Third, heat treatment can be applied to control the final microstructure. For future work, it is suggested that heat treatment (normalizing, quenching, and low temperature tempering) could be applied to the welds in order to better control the microstructure in terms of the phases and having no incompatibilities, such as cracks. Moreover, properties such as hardness and tensile strength could be enhanced. The variation of microstructures in WZ and HAZ can be reduced. Their differences compared to BM can be reduced.

## Figures and Tables

**Table 1:** Chemical composition (wt%) of BM and filler metals used in this research.

		C	Si	Mn	Cr	Mo	Ni	S	Al	Cu	Ti	Co	V	Fe
**Base metal**	**Martensitic**	0.272	0.470	1.180	0.150	0.030	0.090	-	-	-	-	-	-	Bal.
**Filler 1** **(ER80S-Ni1)**	**Ferritic + Austenitic**	0.191	0.517	1.157	0.136	0.039	0.435	0.008	0.029	0.065	0.006	0.007	0.018	Bal.
**Filler 2** **(ER80S-G)**	**Ferritic**	0.194	0.614	1.378	0.100	0.027	0.077	0.011	0.025	0.090	0.006	0.003	0.019	Bal.
**Filler 3** **(ER80S-B2)**	**Austenitic**	0.208	0.508	1.171	0.743	0.312	0.080	0.008	0.034	0.114	0.007	0.004	0.019	Bal.

## Data Availability

Not applicable.
